# 2631. The impact of the COVID-19 pandemic on seasonal respiratory virus circulation in Korean children

**DOI:** 10.1093/ofid/ofad500.2243

**Published:** 2023-11-27

**Authors:** Kyo Jin Jo, Yoon Ha Hwang, Yechan Kyung, Ju-Suk Lee, Su Eun Park

**Affiliations:** Pusan National University Children's Hospital, Yangsan si, Kyongsang-namdo, Republic of Korea; Busan St. Mary’s Hospital, Nam gu, Pusan-jikhalsi, Republic of Korea; Samsung Changwon Hospital, Changwon si, Kyongsang-namdo, Republic of Korea; Samsung Changwon Hospital, Changwon si, Kyongsang-namdo, Republic of Korea; Pusan National University Children's Hospital, Yangsan si, Kyongsang-namdo, Republic of Korea

## Abstract

**Background:**

We aimed to compare the changes in seasonal respiratory viruses in the southern part Korean children between before pre-pandemic seasons (March 2018 to February 2020) and pandemic seasons (March 2020 to February 2022).

**Methods:**

We retrospectively reviewed medical records of hospitalized children (0 to 18 years old) in whom seasonal respiratory viruses were detected using multiplex reverse transcriptase-polymerase chain reaction amplification in three centers from March 2018 to February 2022.

**Results:**

Overall, 7,226 patients were enrolled. There were 6,090 patients in pre-pandemic seasons and 1,136 patients in pandemic seasons. Human rhinovirus and human adenovirus were continuously detected even after COVID-19 outbreak, there was one sample that influenza virus was detected. Parainfluenza virus (PIV) and respiratory syncytial virus (RSV) were almost disappeared in 2020. The PIV was detected from March to December in pre-pandemic seasons, and it was mainly prevalent from May to July. However, after the COVID-19 outbreak, in 2021, only PIV3 was detected from August to December, and it was mainly prevalent in September and October. The RSV was detected from August or September to May or June in pre-pandemic seasons, and it was mainly prevalent from October to February. However, after COVID-19 outbreak, in 2021, RSV was detected from November to February, and it was mainly prevalent in December and February. In PIV3 and RSV, comparing pre-pandemic seasons and pandemic seasons, patients in the pandemic seasons were older and the duration of hospital admission was longer in pre-pandemic seasons than in pandemic seasons. There were no significant differences in the history of underlying chronic diseases and clinical severity in PIV3 and RSV patients.

Monthly distribution of seasonal respiratory viruses

**Figure:**
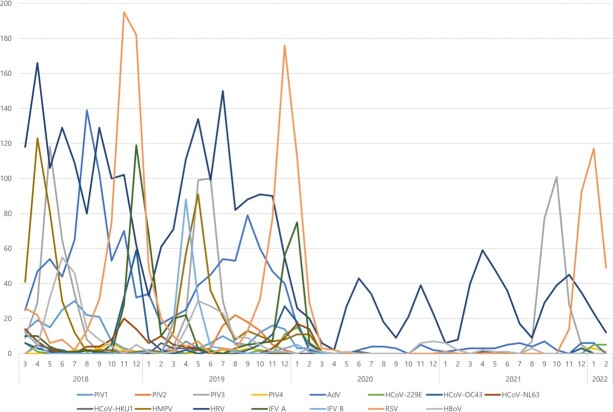

In pre-pandemic seasons, PIVs and HMPV were most commonly detected in spring and early summer, HBoV in late spring and early summer, RSV in October and winter, and IFV and HCoVs in winter. HRV (30.4%) was the most common virus detected, followed by HAdV (16.0%), RSV (15.0%) and HMPV (8.0%). In pandemic seasons, HRV, HAdV, and HBoV were continuously detected even after COVID-19 outbreak, other viruses were rarely detected until the summer of 2021. There was one sample that detected IFV A, and there were no samples that detected PIV1, HCoV-NL63, and IFV B. HRV (50.1%) was the most common virus detected, followed by RSV (22.2%), PIV3 (17.5%), and HAdV (5.5%).

Monthly distribution of PIV3 and RSV

**Figure:**
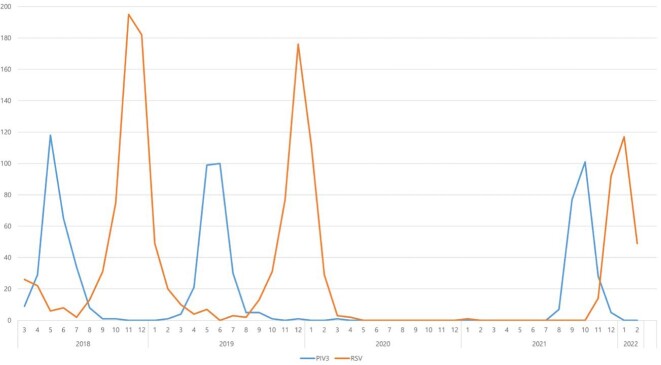

In pandemic seasons, PIV3 and RSV were almost disappeared in 2020. The PIV3 was mainly prevalent from May to July in pre-pandemic seasons, after the COVID-19 outbreak, in 2021, PIV3 was mainly prevalent in September and October. The RSV it was mainly prevalent from October to February in pre-pandemic seasons, however, after COVID-19 outbreak, in 2021, RSV was mainly prevalent in December and February.

**Conclusion:**

The incidence of seasonal respiratory viruses decreased during pandemic seasons, and the prevalent pattern also changed. As the COVID-19 outbreak continues, it is necessary to observe whether changes in seasonal respiratory virus circulation continue.

**Disclosures:**

**All Authors**: No reported disclosures

